# Supplementary material for: Plasma and Urine Metabolites Associated with Microperimetric Retinal Sensitivity in Age-Related Macular Degeneration
Source: Metabolites. 2025 Mar 28;15(4):232. doi: 10.3390/metabo15040232 (PMC12029753; doi:10.3390/metabo15040232)

Supplementary Material

Plasma and Urine Metabolites Associated with Microperimetric Retinal Sensitivity in Age-Related Macular Degeneration

Krupa Sourirajan, Kevin Mendez, Ines Lains, Gregory Tsougranis, Haemin Kang, Georgiy Kozak, Augustine Bannerman, Roshni Bhat, Hanna Choi, Archana Nigalye, Ivana K. Kim, Demetrios G. Vavvas, David M. Wu, Liming Liang, John B. Miller, Joan W. Miller, Jessica Lasky-Su, Deeba Husain

Table of Contents

Figure S1. Schematic Study Workflow.

Figure S2. Principal Component Analysis (PCA) plot of PC1 versus PC2 for plasma samples in Boston.

Figure S3. Principal Component Analysis (PCA) plot of PC1 versus PC2 for urine samples in Boston.

Table S1. Demographic characterization for study participants with urine samples.

Table S2. Plasma metabolites associated with mean retinal sensitivity for All Patients.

Table S3. Plasma metabolites associated with mean retinal sensitivity Stratified by Controls.

Table S4. Plasma metabolites associated with mean retinal sensitivity Stratified by AMD.

Table S5. Urine metabolites associated with mean retinal sensitivity for All Patients.

Table S6. Urine metabolites associated with mean retinal sensitivity Stratified by Controls.

Table S7. Urine metabolites associated with mean retinal sensitivity Stratified by AMD.

*Supplementary Tables are provided on separate sheets within the 'Supplementary for Microperimetry KS.xslx' file.

Figure S1. Schematic Study Workflow.

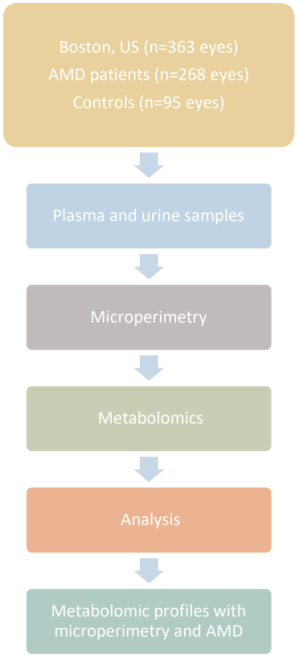

Figure S2. Principal Component Analysis (PCA) plot of PC1 versus PC2 for plasma samples in Boston.

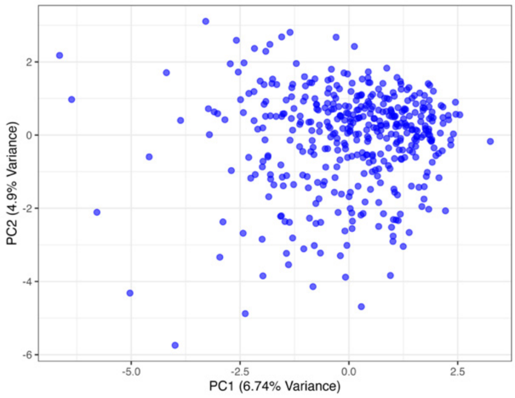

Figure S3. Principal Component Analysis (PCA) plot of PC1 versus PC2 for urine samples in Boston.

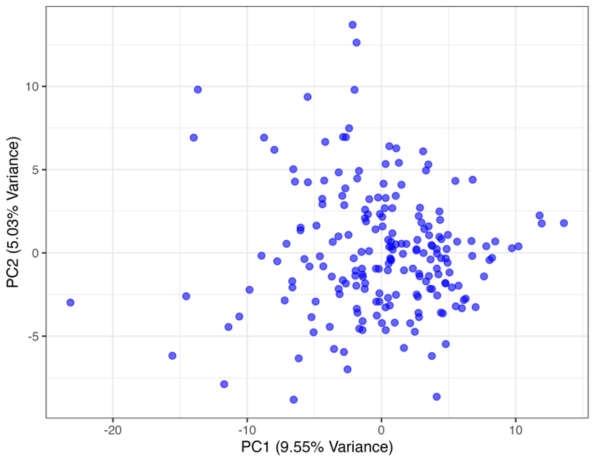

Supplement: Supplementary file 1 [file metabolites-15-00232-s001.zip › Supplementary Material Microperimetry KS 3.10.25 KS.pdf]
